# Mixed metal zero-mode guides (ZMWs) for tunable fluorescence enhancement†

**DOI:** 10.1039/c9na00641a

**Published:** 2020-03-25

**Authors:** Abdullah Al Masud, W. Elliott Martin, Faruk H. Moonschi, So Min Park, Bernadeta R. Srijanto, Kenneth R. Graham, C. Patrick Collier, Christopher I. Richards

**Affiliations:** Department of Chemistry, University of Kentucky Lexington KY 40506 USA chris.richards@uky.edu; Department of Physiology, School of Medicine, University of Kentucky KY 40506 USA; Department of Chemical and Materials Engineering, Univ. of Kentucky 40506 USA; Center for Nanophase Materials Sciences, Oakridge National Lab Oakridge TN 37831 USA

## Abstract

Zero-mode waveguides (ZMWs) are capable of modifying fluorescence emission through interactions with surface plasmon modes leading to either plasmon-enhanced fluorescence or quenching. Enhancement requires spectral overlap of the plasmon modes with the absorption or emission of the fluorophore. Thus, enhancement is limited to fluorophores in resonance with metals (*e.g.* Al, Au, Ag) used for ZMWs. The ability to tune interactions to match a wider range of fluorophores across the visible spectra would significantly extend the utility of ZMWs. We fabricated ZMWs composed of aluminum and gold individually and also in mixtures of three different ratios, (Al : Au; 75 : 25, 50 : 50, 25 : 75). We characterized the effect of mixed-metal ZMWs on single-molecule emission for a range fluorophores across the visible spectrum. Mixed metal ZMWs exhibited a shift in the spectral range where they exhibited the maximum fluorescence enhancement allowing us to match the emission of fluorophores that were nonresonant with single metal ZMWs. We also compared the effect of mixed-metal ZMWs on the photophysical properties of fluorescent molecules due to metal–molecule interactions. We quantified changes in fluorescence lifetimes and photostability that were dependent on the ratio of Au and Al. Tuning the enhancement properties of ZMWs by changing the ratio of Au and Al allowed us to match the fluorescence of fluorophores that emit in different regions of the visible spectrum.

## Abstract 2

Mixed metal ZMWs tune single molecule fluorescence enhancement.

## Graphical abstract

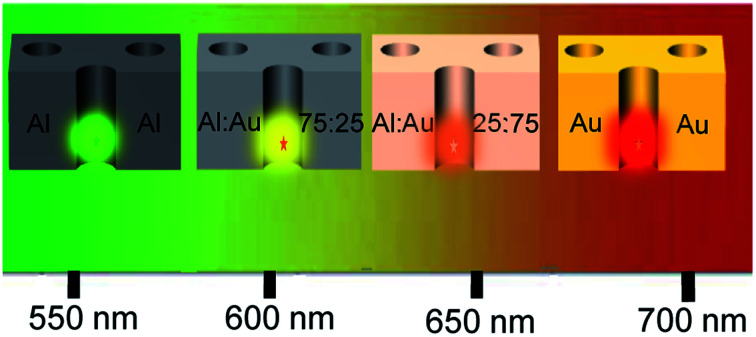

## Introduction

Single molecule spectroscopy is widely used for biological applications including single particle tracking, protein folding, and protein–protein interactions.^1^ However, fluorophore brightness and photostability remain significant challenges for single molecule imaging primarily due to an inability to differentiate signals from background. Additionally, the diffraction limit of excitation light requires the use of nanomolar or lower concentrations to discriminate single-molecule emission from background.^2^ This concentration barrier is problematic because many biomolecules exist at physiological concentrations in the micro to millimolar range. A variety of nanophotonic devices have been developed to address these issues including near-field scanning optical microscopes^3,4^ and zero-mode wave guides (ZMWs).^5^ ZMWs are composed of arrays of nanoscale (<250 nm) holes in thin metal films deposited on a glass substrate. While confocal and total internal reflection (TIRF) modalities are limited to a concentration barrier (<10 nM) to detect a single molecule event,^6^ nanoapertures allow imaging of biological interactions at single molecule levels in micromolar concentrations.^7–15^ As a result, ZMWs have been widely used for genomic sequencing,^16–18^ protein–protein interaction,^19–22^ ligand-receptor binding,^23–28^ membrane bound diffusion events^29,30^ and the study of membrane proteins at single molecule levels.^31,32^ This capability is due to the aperture dimensions of the ZMWs, which result in cut-off wavelengths for the transmission of light smaller than the wavelength of excitation light. Thus, excitation light does not propagate through the wells but creates an evanescent wave at the entrance of the wells that limits the excitation volume to regions just inside the aperture.^33,34^ The photophysical properties of fluorophores isolated in ZMWs can also be altered through interactions with the metal structure. These interactions can affect both excitation and emission processes. Enhanced local fields in and near ZMW wells increase the excitation rate leading to brighter emission and faster photobleaching.^2,35–37^ Additionally, excited fluorophores can interact with surface plasmons resulting in energy transfer to the metal and quenching of the fluorescence signal or coupling with surface plasmons that results in re-radiation, an increase in fluorescence intensity, and shorter fluorescence lifetimes.^38^ Like other metal nanostructures, fluorescence enhancement by nano apertures depends on the size and shape^39–41^ of the nanoholes, metal composition,^39,40,42^ excitation wavelength^35^ and spatial position of fluorophores relative to the metal structures.^7,43,44^ Fluorophores in close proximity to the metal wall experience higher excitation rates, decay rate enhancement, and quenching due to the dissipation of photon energy to the metal wall as heat.^40^

Plasmon mediated fluorescence enhancement by metal nanostructures is also dependent on the spectral overlap of the surface plasmon resonance (SPR) with the excitation and emission spectra of the fluorophores.^45–49^ Many fluorophores have excitation and emission maxima that coincide with the SPR for pure metals such as gold and aluminum. However, commonly-used fluorophores in biological applications have emission spectra that span the visible spectra into the near infrared. There are limited options for nanophotonics capable of plasmon enhanced fluorescence for many of these fluorophores; the ability to tune the SPR to match the fluorophore excitation and emission spectra will extend the range of plasmon-enhanced fluorescence applications. Although the SPR can be shifted to some extent by controlling parameters like particle size and shape, particle-to-particle distance, and surrounding dielectric media,^45,50,51^ the tunability is limited. Mixed-metal ZMWs offer additional degrees of freedom through atomic composition and arrangement.^45,52^ Nanostructures composed of mixtures of metals have been used to create new, dephased plasmon modes, which were different from structures composed of individual metals.^53^ Mixed metals sometime exhibit a broadband SPR which facilitates multi-analyte detection.^54^ Mixed-metal induced fluorescence enhancement in bulk samples has been shown in Silver island Films (SiF) coated with thin aluminum layer,^53^ silver–aluminum nanoislands^54^ and silver–copper nanoparticles.^45^ Additionally, layered Al–Au metal nanophotonic devices have been utilized for single molecule applications.^55,56^

To take advantage of these mixed metal features, we fabricated ZMWs composed of mixtures of aluminum (Al) and gold (Au) at three different ratios; Al : Au (75 : 25, 50 : 50 & 25 : 75). We compared the effect of these mixed metal ZMWs (75Al, 50Al, 25Al) as well as pure Al (100Al) and Au (100Au) devices on the photophysical properties of a series of fluorophores that emitted in different regions of the visible spectra. These devices show tunability across the visible region in their ability to enhance single molecule fluorescence.

## Results and discussion

We fabricated ZMWs with varying compositions of aluminum and gold with identical dimensions, composed of 200 nm diameter holes drilled in a 100 nm metal layer (Fig. S1†). All ZMWs were fabricated by electron beam lithography (EBL) and a lift off process as detailed in the methods. The only fabrication process that differs between single metal ZMWs (100Al and 100Au) and mixed metal ZMWs (75Al, 50Al and 25Al) is the metal layer deposition step. For mixed metal ZMWs both Al and Au were evaporated simultaneously whereas only Al or only Au was evaporated for single metal ZMWs.

**Fig. 1:**
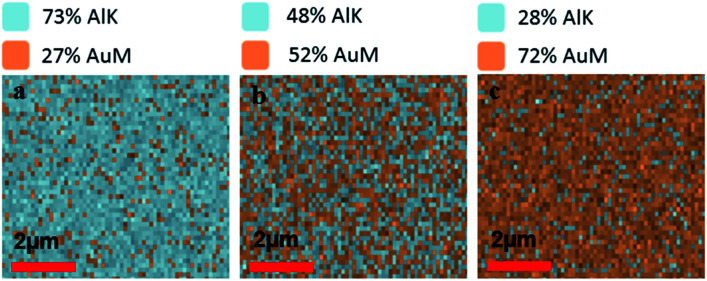
(a–c) Energy dispersive spectroscopy (EDS) images representing the ratio of AlK/AuM emission from the surface of mixed metal ZMWs *i.e.* 75Al, 50Al and 25Al respectively. The ratio of Alk/AuM on the ZMW surfaces matches the target ratio of Al and Au in respective mixed metal ZMWs.

To verify the composition of Al and Au in mixed metal ZMWs, we performed elemental analysis by energy dispersive spectrometer (EDS). Fig. 1a–c show the atomic ratio of Al and Au in 75Al, 50Al and 25Al. The observed composition matched the deposition composition of Al and Au in hybrid ZMWs. We also checked the quality of ZMW wells and found an array of round shaped ZMW wells free from trace of resist polymer (Fig. S2†). To determine the effect of the mixed metal structures on surface plasmons in a thin film, we measured the reflectance spectra of ZMWs in bulk (Fig. S3†). The reflectance spectra of 100Al and 100Au matched that previously reported for pure Al and Au thin films, respectively.^57–59^ While the unique environment in a ZMW aperture would exhibit a different plasmon resonance than that of a thin film, the thin film measurements serve to verify that optical properties change with metal composition. We observed a clear red shift in the surface plasmon in mixed metal ZMWs with increased proportion of Au. This verifies that the mixture of metals shifts the optical properties in mixed metal ZMWs. Mixtures of different ratios of metals have been shown to shift the SPR of nanostructures due to changes in the electrical conductivity.^45,60^ Spectral overlap between the SPR of metal nanostructures and the fluorophore spectrum is the key component for metal enhanced fluorescence, thus shifts in SPR lead to overlap with different spectral regions. The observed changes in the spectral region generating the maximum enhancement measured using a series of fluorophores indicates a shift in SPR. This coincides well with other mixed metal nanostructures where a clear shift was observed in SPR with different ratios of mixed metals. To determine the effect of hybrid ZMWs on fluorophore photophysical properties, all five ZMW types; (100Al, 75Al, 50Al, 25Al and 100Au) were tested with each of four different fluorophores that emit across the visible spectrum (ATTO 550, ATTO 590, ATTO 610, and ATTO 647N). Fluorophores were immobilized in ZMWs using biotin-NeutrAvidin linker chemistry (Fig. S4†). We used an inverted microscope equipped with a piezoelectric stage for all single molecule experiments.

**Fig. 2:**
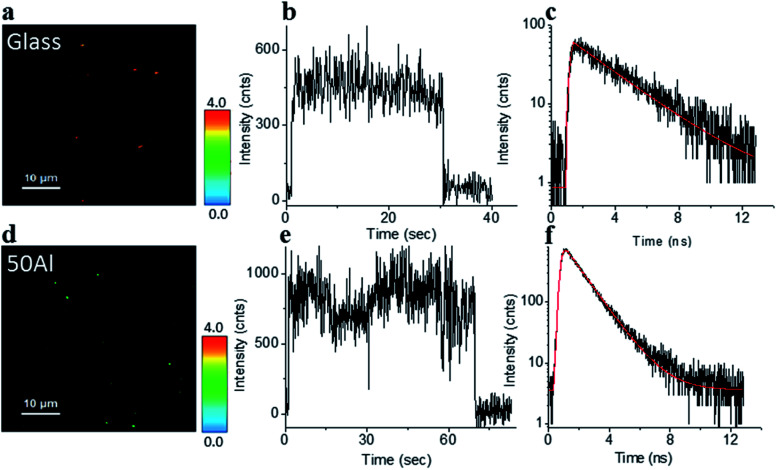
(a and d) Fluorescence lifetime images of single ATTO 550 molecules isolated on glass and on the glass bottom of 50Al respectively, a clear difference in color scale, among the molecules between glass and 50Al indicates a reduction of the fluorescence lifetime of the molecules in 50Al. (b and e) Representative fluorescence intensity time trace of a single ATTO 550 molecule isolated on glass and on the glass bottom of 50Al respectively. (c and f) Fluorescence decay histogram of the same ATTO 550 molecules isolated on glass and on the glass bottom of 50Al respectively.

The stage was raster scanned over a 30 μm × 30 μm to 50 μm × 50 μm area to locate the fluorophores immobilized within this area (Fig. 2a and d). Bright spots in the image represent the location of fluorophores. Fig. 2b and e shows representative fluorescence intensity time traces of single ATTO 550 molecules immobilized on glass and in 50Al, respectively. The observed single step photo bleaching is indicative of single molecule fluorescence. We simultaneously recorded the fluorescence lifetime of individual fluorophores. A clear contrast in color scale among the fluorophore molecules on the glass coverslip *versus* those in 50Al wells (Fig. 2a and d), indicates the shortening of the fluorescence lifetime due to interactions of fluorophores with the ZMW. Fig. 2c and f shows the fitted fluorescence lifetime decay of single fluorophores on a glass coverslip and in 50Al well, respectively. Similar measurements were performed to extract single molecule photophysical characteristics across all ZMW compositions for each of the fluorophores.

### Fluorescence enhancement of ATTO 550

To investigate the wavelength dependence of ZMWs on single molecule fluorescence, we compared single molecule fluorescence properties of ATTO 550 in the five different ZMWs. ATTO 550 molecules immobilized on glass were used as a reference to determine the effects of the plasmonic structures on single molecule fluorescence. The fluorescence intensity time trace of ∼100 single ATTO 550 fluorophores on glass and in all ZMW types were recorded at 532 nm laser excitation (1.03 μW).

**Fig. 3:**
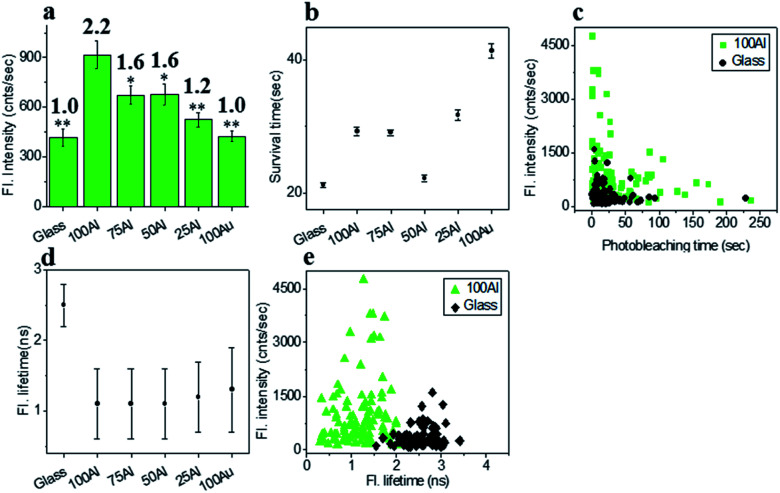
(a) Average fluorescence intensity of single ATTO 550 molecules isolated on glass and on the glass bottom of 100Al, 75Al, 50Al, 25Al and 100Au. The fluorescence enhancement for each ZMW is shown on top of the respective column which is calculated by direct comparison with glass. * indicates *P* < 0.05, ** indicates *P* < 0.001. (b) The survival time of single ATTO 550 molecules isolated on glass and on the glass bottom of 100Al, 75Al, 50Al, 25Al and 100Au. The survival time of single molecules in each substrate is calculated by fitting the photobleaching time of isolated molecules with a single exponential decay. (c) Scatter plot comparison of fluorescence intensity *versus* photobleaching time of single ATTO 550 molecules in 100Al (green) *versus* glass (black). (d) The average fluorescence life time of single ATTO 550 molecules isolated on glass and on the glass bottom of 100Al, 75Al, 50Al, 25Al and 100Au. (e) Scatter plot comparison of fluorescence intensity *versus* fluorescence lifetime of single ATTO 550 molecules in 100Al (green) *versus* glass (black).

The fluorescence intensity of each single fluorophore was then extracted from the respective fluorescence intensity time trace and the resulting fluorescence intensities were histogrammed (Fig. S5†). The average fluorescence intensities in all substrates were compared in Fig. 3a. Enhancement values on top of each bar were calculated by direct comparison with that of glass. It is evident that 100Al gives the maximum fluorescence intensity (2.2 times glass), whereas it is lowest for 100Au (identical to glass). Fluorescence intensity enhancement values for molecules in 75Al, 50Al and 25Al are 1.6, 1.6 and 1.2 times to glass, respectively. There is a clear decreasing trend in fluorescence intensity of single ATTO 550 molecules with increasing ratios of Au, which indicates 100Al is a better match for ATTO 550 than other ZMWs. Molecules isolated in 75Al and 50Al showed similar fluorescence intensities. Fluorescence intensity enhancement in ZMWs might result from increased local excitation rates, increased radiative decay rate, or improved detection efficiency by directing more emission toward the detector. To understand which processes were involved, we simultaneously determined the photostability (survival time) of single molecules isolated in each of the ZMW compositions. The photostability of single emitters was calculated by fitting the photobleaching time of emitters with single exponential decay (Fig. S6†). The average photostability of single emitters on glass and in all ZMWs is shown in Fig. 3b. The average survival time of single ATTO 550 molecules on glass is 21.2 ± 0.4 s whereas the average survival times in 100Al, 75Al, 50Al, 25Al and 100Au were found to be 29.3 ± 0.6 s, 29.1 ± 0.4 s, 22.2 ± 0.5 s, 31.8 ± 0.8 s and 41.4 ± 1.1 s respectively. This indicates that photostability increased in all ZMWs compared to glass but was highest for 100Au (∼2.0 times glass) and lowest for 50Al (similar to glass). Enhancement in fluorescence intensity coupled with improved photostability for molecules in 100Al, 75Al and 25Al could be an indication of both excitation and decay rate enhancement. Increasing only the excitation rate through interactions with the localized electric field increases the average fluorescence signal but also leads to more rapid photobleaching. However, interactions of excited fluorophores with ZMWs create alternative decay pathways, which compete with transitions that lead to photobleaching, resulting in improved photostability. The enhanced fluorescence intensity with no apparent change in photostability for the molecules in 50Al indicates a combination of factors likely arising from excitation side enhancement and coupling into an alternative relaxation pathway that reduces the likelihood of photobleaching. Maximum enhancement in the photostability but no enhancement in fluorescence intensity for molecules in 100Au suggests decay rate enhancement through a non-radiative pathway. Alternatively, a scatter plot comparison of photobleaching time *versus* fluorescence intensity of individual ATTO 550 molecules suggest that a larger proportion of molecules in 100Al are brighter and more photostable than the molecules on glass (Fig. 3c). However, it should be mentioned that there is a large distribution of fluorescence intensity and photobleaching time values among individual molecules. Spatial localization of molecules within the circular area of the glass bottom of ZMW holes of 200 nm diameter play crucial rule in interactions with the enhanced local field.^44^ Molecules residing several nanometers away from the metal nanostructure experience the highest local excitation field.^61^ To further investigate the mechanism of fluorescence enhancement by ZMWs, we also measured fluorescence lifetimes of single ATTO 550 molecules in each substrate. Histograms are shown in Fig. S7.† Average fluorescence lifetimes of the molecules in all substrates are presented in Fig. 3d. The average fluorescence lifetime of molecules on glass is 2.5 ± 0.4 ns whereas the average values are 1.1 ± 0.5 ns, 1.1 ± 0.5 ns, 1.1 ± 0.5 ns, 1.2 ± 0.5 ns and 1.3 ± 0.6 ns for molecules for 100Al, 75Al, 50Al, 25Al and 100Au respectively. This indicates that the fluorescence lifetime is decreased in all ZMWs. Shortened fluorescence lifetimes are indicative of increased decay rates which might be radiative or nonradiative. The enhanced fluorescence intensity and improved photostability but shortening in fluorescence lifetime for molecules in 100Al, 75Al and 25Al provides further evidence of enhancement by increased decay rate. However, the fluorescence lifetime is also decreased for molecules in 50Al which suggests an enhanced decay rate. The decreased fluorescence lifetime and improved photostability for molecules in 100Au, suggests decay rate enhancement. Coupled with the lack of a significant increase in fluorescence intensity this indicates the presence of mostly nonradiative decay pathways. A plot of the fluorescence intensity *versus* fluorescence lifetime of individual ATTO 550 molecules (Fig. 3e), indicates that the population of molecules in 100Al have shorter lifetimes but are brighter than those on glass.

### Fluorescence enhancement of ATTO 590

**Fig. 4:**
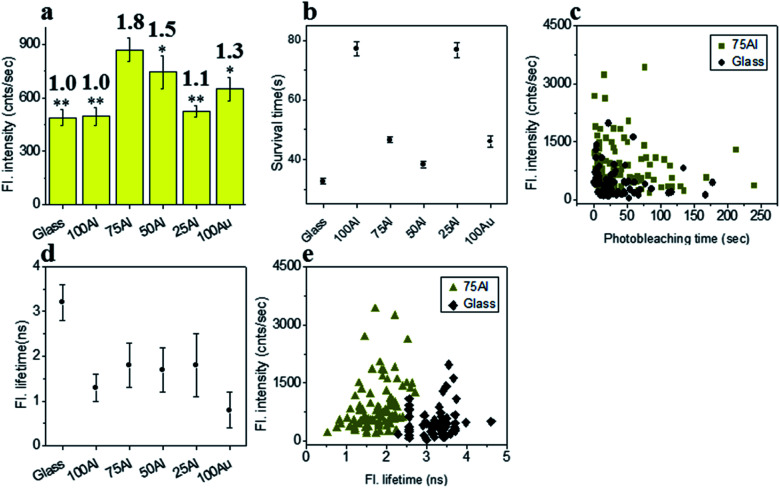
(a) Average fluorescence intensity of single ATTO 590 molecules isolated on glass and on the glass bottom of 100Al, 75Al, 50Al, 25Al and 100Au. The fluorescence enhancement for each ZMW is shown on top of the respective column which is calculated by direct comparison with glass. * indicates *P* < 0.05, ** indicates *P* < 0.001. (b) The survival time of single ATTO 590 molecules isolated on glass and on the glass bottom of 100Al, 75Al, 50Al, 25Al and 100Au. The survival time of single molecules in each substrate is calculated by fitting the photobleaching time of isolated molecules with a single exponential decay. (c) Scatter plot comparison of fluorescence intensity *versus* photobleaching time of single ATTO 590 molecules in 75Al (gold) *versus* glass (black). (d) The average fluorescence lifetime of single ATTO 590 molecules isolated on glass and on the glass bottom of 100Al, 75Al, 50Al, 25Al and 100Au. (e) Scatter plot comparison of fluorescence intensity *versus* fluorescence lifetime of single ATTO 590 molecules in 75Al (gold) *versus* glass (black).

To determine if the increased ratio of Au led to better enhancement in molecules with red shifted emission, we measured the enhancement in each ZMW with ATTO 590 by comparing single molecule fluorescence. 75Al induced the maximum fluorescence enhancement (1.8 fold compared to glass) while the lowest was observed in 100Al (identical to glass) (Fig. 4a and S8†) indicating the best congruence between 75Al and ATTO 590. Enhancement of the fluorescence intensity of ATTO 590 molecules in 50Al, 25Al and 100Au are 1.5, 1.1 and 1.3 times glass, respectively. There is also a decreasing trend in fluorescence intensity of single ATTO 590 molecules from 75Al to 100Au that signifies a red shift in the compatibility of ZMWs with increasing Au content. Similar to ATTO 550, the photostability of single ATTO 590 molecules also increased in all ZMWs (Fig. 4b and S9†) indicating enhancement of the fluorescence intensity by both increased excitation and decay rates. The magnitude of the improvement in photostability varied among ZMWs. The increase in photostability was greatest in 25Al (2.4 times to glass) and lowest in 100Au (1.3 times to glass). A scatter plot of the fluorescence intensity *versus* photostability of individual ATTO 590 molecules also shows that a large fraction of molecules in 75Al are brighter and more photostable than those on glass (Fig. 4c). Similar to ATTO 550, the fluorescence lifetime of ATTO 590 decreased from 3.2 ± 0.4 ns on glass to below 1.8 ± 0.8 ns (Fig. 4d and S10†) for all ZMWs which is an indication of enhanced decay rates. Although the lifetime was shortened in all ZMWs, ATTO 590 molecules in 100Al and 25Al exhibited a lower average fluorescence intensity than the other ZMWs with values similar to glass. This suggests that both radiative and non-radiative alternative decay pathways are present. Alternatively, the observed maximum enhancement in fluorescence intensity coupled with shorter fluorescence lifetimes for ATTO 590 molecules in 75Al indicates radiative decay pathways account for the majority of the increased decay rate. A comparison of the fluorescence intensity *versus* fluorescence lifetime of individual ATTO 590 molecules also shows that 75Al induced shorter lifetimes and enhanced fluorescence intensity compared to molecules on glass (Fig. 4e).

### Fluorescence enhancement of ATTO 610

**Fig. 5:**
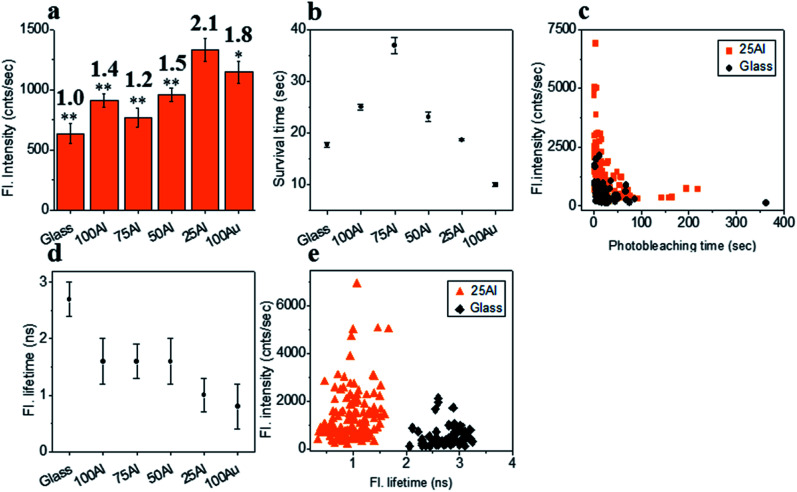
(a) Average fluorescence intensity of single ATTO 610 molecules isolated on glass and on the glass bottom of 100Al, 75Al, 50Al, 25Al and 100Au. The fluorescence enhancement for each ZMW is shown on top of the respective column which is calculated by direct comparison with glass. * indicates *P* < 0.05, ** indicates *P* < 0.001. (b) The survival time of single ATTO 610 molecules isolated on glass and on the glass bottom of 100Al, 75Al, 50Al, 25Al and 100Au. The survival time of single molecules in each substrate is calculated by fitting the photobleaching time of isolated molecules with a single exponential decay. (c) Scatter plot comparison of fluorescence intensity *versus* photobleaching time of single ATTO 610 molecules in 25Al (orange) *versus* glass (black). (d) The average fluorescence life time of single ATTO 610 molecules isolated on glass and on the glass bottom of 100Al, 75Al, 50Al, 25Al and 100Au. (e) Scatter plot comparison of fluorescence intensity *versus* fluorescence lifetime of single ATTO 610 molecules in 25Al (orange) *versus* glass (black).

We also tested ATTO 610 in each of the ZMWs. The maximum fluorescence intensity (2.1 fold compared to glass) for single ATTO 610 molecules was in 25Al (Fig. 5a and S11†). This is a further indication of the red shifting of the compatibility of the ZMW with increased Au content. The fluorescence intensity was also enhanced for molecules in other ZMWs with 1.4, 1.2, 1.5 and 1.8 times for 100Al, 75Al, 50Al and 100Au, respectively. The photostability increased for single ATTO 610 molecules in 100Al, 75Al and 50Al which showed increases over glass of 1.5, 2.1 and 1.3-fold, respectively. While the photostability was similar to glass for 25Al, it decreased by 44% for 100Au (Fig. 5b and S12†). Enhanced fluorescence intensity with improved photostability for molecules in 100Al, 75Al and 50Al indicates both excitation and decay rate enhancement. Enhanced fluorescence intensity coupled with decreased photostability in 100Au indicates excitation rate enhancement. A comparison of the fluorescence intensity *versus* photostability of individual ATTO 610 molecules shows that a large fraction of molecules in 25Al are brighter than those on glass (Fig. 5c). The fluorescence lifetime of single ATTO 610 molecules decreased in all ZMWs (Fig. 5d and S13†). The average fluorescence lifetime of molecules on glass was 2.7 ± 0.3 ns, whereas the average fluorescence lifetime of molecules in 100Al, 75Al, 50Al, 25Al and 100Au was 1.6 ± 0.4 ns, 1.6 ± 0.3 ns, 1.6 ± 0.4 ns, 1.0 ± 0.3 ns and 0.9 ± 0.4 ns, respectively. The shortened fluorescence lifetime with improved photostability for molecules in 100Al, 75Al and 50Al indicates decay rate enhancement. Furthermore, fluorescence intensity also increased for molecules in these ZMWs which suggests the decay pathways are radiative. Enhanced fluorescence intensity coupled with decreased fluorescence lifetime but no significant changes in photostability for molecules in 25Al suggests a combination of excitation side and decay rate enhancement. In the case of molecules in 100Au, the fluorescence intensity is increased with reduced photostability which is a signature of excitation enhancement. The fluorescence lifetime is also reduced which indicates decay rate enhancement. Molecules in 100Au likely exhibit both excitation and decay rate enhancement, but the excitation rate enhancement is more prominent than the decay rate which ultimately shortened the photostability. A molecule by molecule comparison of ATTO 610 in 25Al *versus* glass plotting fluorescence intensity *versus* fluorescence lifetime shows that molecules in 25Al are shorter in lifetime but brighter than those of glass (Fig. 5e).

### Fluorescence enhancement of ATTO 647N

**Fig. 6:**
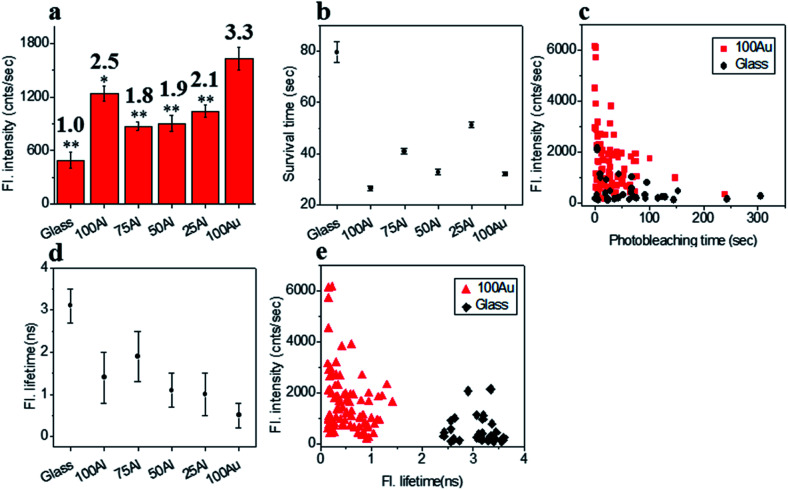
(a) Average fluorescence intensity of single ATTO 647N molecules isolated on glass and on the glass bottom of 100Al, 75Al, 50Al, 25Al and 100Au. Fluorescence enhancement for each ZMW is shown on top of the respective column which is calculated by direct comparison with glass. * indicates *P* < 0.05, ** indicates *P* < 0.001. (b) The survival time of single ATTO 647N molecules isolated on glass and on the glass bottom of 100Al, 75Al, 50Al, 25Al and 100Au. Survival time of single molecules in each substrate is calculated by fitting the photobleaching time of isolated molecules with single exponential decay. (c) Scatter plot comparison of fluorescence intensity *versus* photobleaching time of single ATTO 647N molecules in 100Au (red) *versus* glass (black). (d) Average fluorescence lifetime of single ATTO 647N molecules isolated on glass and on the glass bottom of 100Al, 75Al, 50Al, 25Al and 100Au. (e) Scatter plot comparison of fluorescence intensity *versus* fluorescence lifetime of single ATTO 647N molecules in 100Au (red) *versus* glass (black).

We also compared the far-red fluorophore ATTO 647N in each of the ZMWs using 640 nm laser excitation. The maximum enhancement in fluorescence intensity was observed in 100Au which was more than three-fold brighter than molecules on glass (Fig. 6a and S14†). The Fluorescence intensity also increased in the other ZMWs with changes in fluorescence of 2.5, 1.8, 1.9 and 2.2 times in 100Al, 75Al, 50Al and 25Al respectively. There is a clear trend of larger enhancement with increasing Au content with the exception that 100Al shows much better fluorescence intensity than expected which could arise from increase in excitation rate. The photostability of single ATTO 647N molecules in all ZMWs decreased (Fig. 6b and S15†). The survival time of ATTO 647N molecules on glass is 79.6 ± 4.1 s, however, the survival times are 26.5 ± 1.0 s, 41.0 ± 1.0 s, 32.8 ± 1.1 s, 51.3 ± 1.0 s and 32.1 ± 0.7 s for molecules in 100Al, 75Al, 50Al, 25Al and 100Au. This indicates that survival time is greatly reduced for molecules in 100Al by 66.7%. The reduced percentage of survival times for molecules in 75Al, 50Al, 25Al and 100Au are 48.5%, 58.8%, 35.6% and 59.7%, respectively. A scatter plot of the fluorescence intensity *versus* photobleaching time of individual ATTO 647N molecules reveals that molecules in 100Au are brighter but less photostable than those on glass (Fig. 6c). Enhanced fluorescence intensity with reduced photostability indicates excitation rate enhancement for molecules in all ZMWs. The fluorescence lifetime decreased for molecules in all ZMWs (Fig. 6d and S16†) which suggests decay rate enhancement. Thus, both excitation and decay rate enhancement likely contribute to the increased fluorescence intensity in all ZMWs. However, decay rate enhancement should result in improved photostability, which was not observed. Both excitation and decay rate enhancement likely contribute to changes in fluorescence count rate. The decrease in photostability indicates that the increased excitation rate plays a more prominent role. A scatter plot of fluorescence intensity *versus* fluorescence lifetime of individual ATTO 647N molecules shows that molecules in 100Au are brighter but have shorter lifetimes than those on glass (Fig. 6e).

### Physical mechanism of fluorescence enhancement by ZMWs

**Fig. 7:**
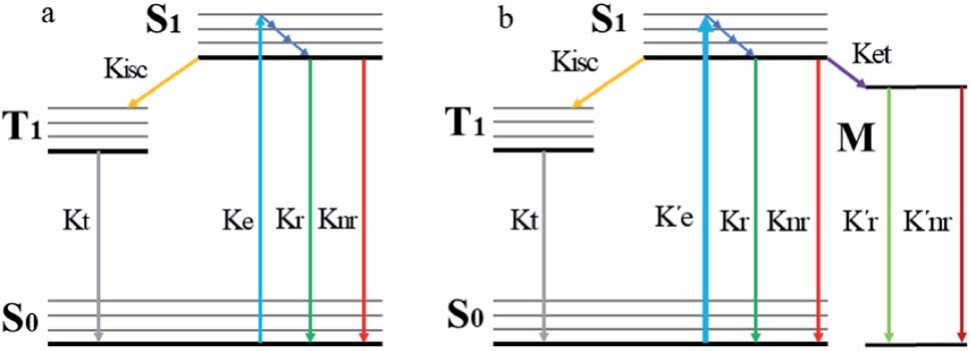
(a) Jablonski diagram in the absences of the ZMW. An electron is promoted from the ground state (S0) to an excited state (S1). The electron rapidly relaxes back from higher vibrational energy levels to lowest vibrational level of first excited state. The electron can relax back to the ground state *via* three intrinsic decay pathways (1) a radiative decay pathway (green arrow) where energy is released as a photon; (2) a non-radiative pathway (red arrow) where energy is lost as heat with no emission; and (3) intersystem crossing where the electron transitions to the triplet state (T1) and ultimately non-radiatively relaxes back to ground state. (b) Jablonski diagram in the presence of the ZMW. In the presence of a plasmonic structure, the excitation rate 
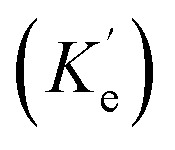
 can be enhanced leading to an increased cycling between the ground and excited states. This leads to an increase in the observed fluorescence and a decrease in the photostability. The presence of alternate decay pathways associated with the plasmonic structure leads to either emissive 
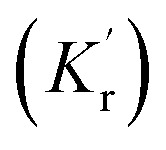
 or nonemissive 
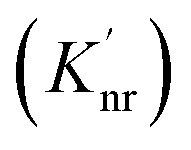
 relaxation from the excited sate. These alternate pathways compete with the traditional relaxation from the excited state (*K*_r_ and *K*_nr_) leading to a reduced fluorescence lifetime.

When a fluorophore is in close proximity to a plasmonic structure like a ZMW, the excitation rate can be increased due to the enhanced electric field generated from surface plasmons. A standard Jablonski diagram is shown in Fig. 7a where excitation of an electron from the ground to the excited state is represented along with the possible decay pathways including through radiative and nonradiative pathways. The inherent fluorescence lifetime results from a combination of these decay pathways.1
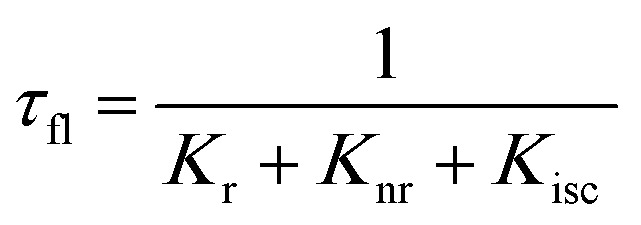


An increase in the excitation rate results in an increase in the cycling of the electron from the ground state to the excited. Excitation side enhancement due to an increase in the excitation rate does not change the decay pathways and does not alter the fluorescence lifetime. The probability of transitioning to a dark state (*e.g.* triplet state) that can lead to photobleaching remains the same. Thus, as the rate of the cycling between the ground and excited state increases, photobleaching occurs on a shorter time scale. Near-field coupling of the excited state of the molecule with surface plasmons leads to new decay pathways that can radiative and nonradiative (Fig. 7b). These alternate relaxation pathways compete with the inherent pathways altering the intrinsic fluorescence lifetime as described in eqn (2). This process also decreases the probability of transition to bleaching prone dark states which results in an increase in the photostability. As a result, interactions with plasmonic particles can either increase or decrease the observed photostability and simultaneously decrease the fluorescence lifetime. We attribute increased emission coupled with reduced photostability with excitation side enhancement. We also attribute increased photostability coupled with decreased fluorescence lifetimes with coupling into new relaxation pathways. It is not clear that observed changes in the interactions with the ZMWs result from a shift in the plasmon resonance. It is clear that mixed metal ZMWs can be tuned to match fluorophores in different spectral regions in order to enhance their emission.2
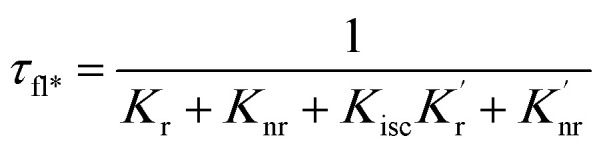


## Experimental

### Nanofabrication of ZMWs

Five different types of ZMWs with different metal composition were fabricated according to published protocols.^2,62^ A metal lift-off process of patterns defined using electron beam lithography was performed at the Center for Nanophase Materials (CNMS) facility at Oakridge National Lab. Cleaned glass coverslips were coated with adhesion promoter Microprimer P-20 (Shin-Etsu MicroSi, Inc.) by spin coating at 2000 rpm for 45 seconds, followed by spin coating of a high resolution negative tone polymer resist, NEB-22A (Sumika Corp.), also at 2000 rpm for 45 seconds. The glass substrates were soft-baked on a hot plate at 110 °C for 2 minutes. ZMW features consist of 200 nm diameter dots, were patterned using JEOL JBX-9300FS E-beam lithography system with a base dose of 80 μC cm^−2^, 100 kV acceleration voltage and 500 pA beam current. After exposure, substrates were post-exposure baked at 95 °C for 4 minutes. Development process was done in Microposit MF-321 solution for 30 seconds followed by rinsing in DI water and drying with nitrogen gas, leaving arrays of evenly spaced pillars of NEB-22A. The glass substrates were then treated with oxygen plasma at 100 W and 10 sccm O_2_ for 6 seconds in an Oxford Plasmalab System 100 Reactive Ion Etcher to remove any residual resist. Metals deposition on the glass substrates was carried out in a dual gun e-beam evaporator. A 5 nm chromium film was used as an adhesion layer followed by a deposition of 100 nm of Al, Au or a mixture of both. Mixed metal ZMWs was achieved by depositing both metals simultaneously at different deposition rates. Lift off process of NEB-22A was performed by submerging the devices in Microposit Remover 1165 (NMP) for 30 minutes at 70 °C in a NMP bath and subsequent sonication for 30 minutes resulted in arrays of round ZMW wells.

### Single fluorophore binding

Single fluorophores were attached to cleaned glass substrates using biotin-NeutrAvidin linker chemistry. Biotin-PEG-Silane (Laysan Bio) at a concentration of 2 mg ml^−1^ in 95% ethanol was allowed to bind on surface for 30 minutes. Following rinsing with 95% ethanol, 100 μM biotin binding protein, NeutrAvidin (Sigma Aldrich) in 1× PBS buffer pH 7.0 was added and allowed to bind for 2 hours. After rinsing thoroughly with 1× PBS buffer pH 7.0, biotin bound fluorophores (ATTO Tech., Germany) in 1× PBS buffer pH 7.0 were allowed to bind for 5 minutes. As NeutrAvidin has 4 binding sites for biotin, non-fluorescent biotin was added with the fluorophore solution to avoid multiple fluorophore binding with a single NeutrAvidin molecule. To avoid nonspecific binding of dye molecules on the wall of ZMWs, a protective coating of PVPA was employed by adding 2% v/v aqueous solution of PVPA (poly(vinylphosphonic acid)) at 110 °C for 2 minutes to the ZMW, followed by a rinsing with DI water and let dry for 10 minutes at 80 °C on a hot plate. As PVPA has a higher binding affinity for metals than glass, it preferentially binds with the metal wall of ZMWs.^63^

### Single molecule data acquisition

Time tagged data acquisition was performed using a confocal microscopy setup equipped with an inverted, Olympus IX 83 microscope. A SuperK Extreme Supercontinuum Free Space Pulsed Laser was used as an excitation source. A piezoelectric stage (Mad City Lab) was employed to raster scan a 30 × 30 to 50 × 50 μm^2^ area of the substrate surface to detect the molecules immobilized on the surface. Excitation light was focused on the substrate through a 60×, 1.45 NA oil immersion objective. Fluorescence emission cleared of back scattered light by a dichroic mirror was passed through a 100 μm pinhole to remove defocused light. An avalanche photodiode detector (APD) was used to detect emitted photon and a time correlated single photon counter (TCSPC) counted single photon events. ATTO 550 molecules were excited at 532 nm filtered through double excitation filters (ZET 532/10X, Chroma), and the emission was passed through an ET 542 LP (Chroma) and ET 575/40M filter (Chroma). The excitation power was 1.03 μW. Both ATTO 590 and ATTO 610 molecules were excited with 594 nm laser light and filtered through a single excitation filter (ZET 594/10X, Chroma), and emission was passed through a HQ 650/75M filter. The laser power for ATTO 590 and ATTO 610 was 0.18 μW and 2.19 μW respectively. ATTO 647N molecules were excited with 640 nm laser light filtered through double excitation filters (ZET 640/10X, Chroma), and the emission was passed through double ET 673/44M filters (Chroma). Laser power was 1.25 μW.

### Data analysis

A custom MATLAB script was used to extract fluorescence intensity time trace data from and plot the fluorescence intensity *vs.* time for each molecule. The average fluorescence intensity of each molecule was calculated by subtracting the average intensity of the time points beyond photobleaching from the average intensity of the time points before bleaching. Survival time of molecules in each substrate was calculated by fitting the photobleaching times of single molecules with single exponential decay. Fluorescence lifetime of each molecule was calculated by fitting the fluorescence lifetime histogram using *n*-exponential deconvolution. Average fluorescence intensity was reported as mean ± SEM, however, both average survival time and fluorescence lifetime were reported as mean ± SD.

## Conclusions

We compared the effect of different ZMW structures on the photophysical properties of fluorophores. This allowed us to determine how ZMW composition altered the properties of fluorophores from different spectral regions. We observed shifts in the spectral regions that exhibited the highest levels of fluorescence enhancement for ZMWs composed of mixtures of Al and Au. 100Al ZMWs yielded the highest level of enhancement for fluorophores that emit at higher energy such as ATTO 550. We also observed that the highest level of enhancement in 100Au occurred with far red to near infrared fluorophores such as ATTO 647N. These shifts at different ratios of Au : Al allowed us to tune interactions to match the emission of fluorophores that emit in the green and orange regions of the visible spectrum. The photostability of ATTO 550 and ATTO 590 molecules in all ZMWs was enhanced which indicates both excitation and emission side fluorescence enhancement. However, the photostability of ATTO 647N molecules in all ZMWs was lowered which suggests the observed fluorescence enhancement most likely resulted from an increase in the excitation rate. Furthermore, fluorescence lifetimes of all fluorophores in every ZMW are significantly decreased which indicates the presence of alternate decay pathways from the excited state through interactions with ZMWs. These studies illustrate that the properties of ZMWs can be tuned across the visible spectrum by mixing the ratios of two metals. It also provides a map for the selection of fluorescence tags for single molecule studies in ZMWs.

## Conflicts of interest

There is no conflict of interest.

## Supplementary Material

NA-002-C9NA00641A-s001
