# Supplementary material for: Mixed metal zero-mode guides (ZMWs) for tunable fluorescence enhancement
Source: Nanoscale Adv. 2020 Mar 25;2(5):1894–903. doi: 10.1039/c9na00641a (PMC9419232; doi:10.1039/c9na00641a)
Supplement: NA-002-C9NA00641A-s001 [file NA-002-C9NA00641A-s001.pdf]

Electronic Supplementary Material (ESI) for Nanoscale Advances.
This journal is © The Royal Society of Chemistry 2020

Electronic supplementary information for

Mixed metal zero-mode guides (ZMWs) for tunable fluorescence enhancement

Abdullah Al Masud[a], W. Elliott Martin[a], Faruk H. Munschi[b], So Min Park[c], Bernadeta R. Srijanto[d], C. Patrick Collier[d], Keeneth R Graham[a], Christopher I. Richards[a*].

[a]Department of Chemistry, University of Kentucky, 505 Rose street, Lexington KY 40506.

[b]Department of Physiology, University of Kentucky, Lexington, KY 40536

[c]Department of Materials Engineering, University of Kentucky, Lexington KY 40506.

[d]Center for Nanophase Materials Sciences, Oakridge National Lab, Oakridge TN 37831.

Nanofabrication of ZMWs

Five different types of ZMWs having different metallic composition were fabricated according to published protocol[1, 2]. A metal lift-off process of patterns defined using electron beam lithography was performed at the Center for Nanophase Materials (CNMS) facility at Oakridge National Lab. Cleaned glass coverslips were coated with adhesion promoter Microprimer P-20 (Shin-Etsu MicroSi, Inc) by spin coating at 2000 rpm for 45 seconds, followed by spin coating of a high resolution negative tone polymer resist, NEB-22A (Sumika Corp.), also at 2000 rpm for 45 seconds. The glass substrates were soft-baked on a hot plate at 110°C for 2 minutes. ZMW features consist of 200nm diameter dots, were patterned using JEOL JBX-9300FS E-beam lithography system with a base dose of 80 $\mu C/cm^2$, 100kV acceleration voltage and 500pA beam current. After exposure, substrates were post-exposure baked at 95ºC for 4 minutes. Development process was done in Microposit MF-321solution for 30 seconds followed by rinsing in DI water and drying with nitrogen gas, leaving arrays of evenly spaced pillars of NEB-22A. The glass substrates were then treated with oxygen plasma at 100W and 10 sccm O2 for 6 seconds in an Oxford Plasmalab System 100 Reactive Ion Etcher to remove any residual resist. Metals deposition on the glass substrates was carried out in a dual gun e-beam evaporator. A 5 nm chromium film was used as an adhesion layer followed by a deposition of 100 nm of Al, Au or a mixture of both. Mixed metal ZMWs was achieved by depositing both metals simultaneously at different deposition rates. Lift off process of NEB-22A was performed by submerging the devices in Microposit Remover 1165 (NMP) for 30 minutes at 70°C in a NMP bath and subsequent sonication for 30 minutes resulted in arrays of round ZMW wells.

Characterization of ZMWs

ZMWs were checked with a FEI-SEM integrated with Energy Dispersive Spectroscopy (EDS) detector, SEM images of ZMWs were taken to check the size and shape of ZMW holes and also to confirm the proper lifting off the resist. Elemental analysis of mixed metal ZMWS was performed with EDS to verify the ratio of Al and Au.

Surface functionalization of glass coverslips

Glass coverslips were cleaned according to the same glass cleaning procedure mentioned in the nanofabrication of ZMWs section and glued to a petri dish by UV-light exposure for 1 hour. Biotin-PEG-Silane at a concentration of 2 mg/ml in 95% ethanol was added to a glass coverslip for 30 minutes and rinsed thoroughly with 1X PBS buffer pH 7.0. 100 µM biotin binding protein neutrAvidin in 1X PBS buffer pH 7.0 was added for 2.0 hours and rinsed thoroughly with 1X PBS buffer pH 7.0.

Surface Functionalization of ZMWs

All five types of ZMWs i.e 100Al, 75Al, 50Al, 25Al and 100Au were cleaned by thorough rinsing with GenPure 18 MΩ pure DI H_2O, 100% ethanol, followed by plasma cleaning with Harrick plasma cleaner for 5 minutes. To avoid nonspecific binding of dye molecules on the wall of ZMWs, a protective coating of PVPA was employed by adding 2% V/V aqueous solution of PVPA (Poly (vinylphosphonic acid)) at 110°C for 2 minutes to the ZMW, followed by a rinsing with DI water and let dry for 10 minutes at 80°C on a hot plate. As PVPA has higher binding affinity for metals than glass, it preferentially binds with the metal wall of ZMW's[3] . PVPA coated ZMWs were then functionalized with biotin-neutrAvidin linker chemistry. 2 mg/ml Biotin-PEG-Silane (Laysan Bio) in 95% ethanol was added to it for 30 minutes, rinsed with 1X PBS buffer 7.0 and 100 µM neutrAvidin (Sigma Aldrich) in 1X PBS buffer 7.0, left idle for 2 hours to promote binding, and finally rinsed with 1X PBS buffer 7.0 to remove any residual unbound neutrAvidin.

Single fluorophore binding. Fluorophore molecules were bound to the surface neutrAvidin by biotin –neutrAvidin linkage (Fig. S2). Four different types of biotin-conjugated fluorophores were used: ATTO 550-biotin, ATTO 590- biotin, ATTO 610-biotin and ATTO 647N-biotin whose fluorescence spectra cover almost the entire visible range. All the fluorophores were purchased from ATTO-tech, Germany. Biotin bound fluorophore molecules were diluted stepwise in 1X PBS buffer 7.0 to achieve single molecule level concentrations. As NeutrAvidin has 4 binding sites for biotin, non- fluorescent biotin was added with the fluorophore solution to avoid multiple fluorophore binding with a single neutrAvidin molecule.

For Glass, 1-10 pM of biotin-bound fluorophore in 1X PBS buffer pH 7.0: 100 nM of biotin in 1X PBS buffer pH 7.0 was added to for 5 minutes and rinsed thoroughly with 1X PBS buffer pH 7.0. For ZMW's, 1-10 nM of biotin bound fluorophore in 1X PBS buffer pH 7.0: 100 nM of biotin in 1X PBS buffer pH 7.0 was added to for 10 minutes and rinsed thoroughly with 1X PBS buffer pH 7.0.

Single molecule data acquisition. Time tagged data acquisition was performed using a custom built confocal microscope setup on an inverted, dual stack Olympus IX-83 microscope frame**.** For the excitation source, a SuperK Extreme Supercontinuum Free Space Pulsed Laser was used. Substrates were placed on a piezo electric stage (Mad City lab) and raster scanned at 30x30µm^2 – 50x50µm^2 area monodirectionally with excitation light with 4 ms dwell time in each pixel. Excitation laser light was filtered by passing it through narrow band pass filter optics to remove stray light and focused on the substrate surface using an Olympus 60X x 1.45 NA oil objective. Emitted light along with some reflected excitation light travel back through objective. A dichroic mirror, however, filters out the reflected excitation light but permits emission light to pass through it. Emission light then passed through a 100 µm pin hole, an emission filter, and finally collected by an avalanche photo diode (APD). The APD converts single photons into an electrical signal, which is time tagged using a photon counter (picoharp 300). The Picoharp 300 communicates with the PC and Symphotime 64 software assigns the photon to each pixel generating an image of the area scanned such that fluorophores appear as bright spots in the image. The Piezo electric nano positioning stage was then used to position the fluorophore in the location of the confocal beam and the excitation beam was unshuttered to expose the fluorophore for at least 10 seconds beyond the time period the molecule photobleached. Fluorescence emission data was analyzed using Symphotime 64 software as a plot of fluorescence intensity time trace and fluorescence lifetime histogram. ATTO 550 molecules were excited with 532 nm laser light and filtered through double excitation filters (ZET 532/10X, chroma), and emission was passed through ET 542 LP (Chroma) and ET 575/40M (Chroma). Excitation power was 1.03 µW. Both ATTO 550 and ATTO 610 molecules were excited with 594 nm laser light filtered through a single excitation filter (ZET 594/10X, chroma), and emission was passed through HQ 650/75M. However, the laser power for ATTO 590 and ATTO 610 was 0.18 µW and 2.19 µW respectively. ATTO 647N molecules were excited with 640 nm laser light filtered through double excitation filters (ZET 640/10X, chroma), and emission was passed through double ET 673/44M (chroma). Laser power was 1.25 µW.

Reflectance. Reflectance spectra were recorded using a 2 inch diameter integrating sphere (Thorlabs) coupled with an Ocean Optics QEPro spectrometer equipped with a thermoelectric cooled CCD detector and an Ocean Optics Deuterium-Tungsten Halogen light source. The substrates were placed against the exit port of the integrating sphere and angled slightly to reduce specular reflection directly back to the entrance port.

Data analysis

A custom MATLAB script was used to extract fluorescence intensity time trace data and plot the fluorescence intensity vs time for each molecule. The average fluorescence intensity of each molecule was calculated by subtracting the average intensity of the time points beyond photobleaching from the average intensity of the time points before bleaching. Survival time of molecules in each substrate was calculated by fitting the photobleaching times of single molecules with single exponential decay. The fluorescence lifetime of each molecule was calculated by fitting the fluorescence lifetime histogram using n-exponential deconvolution. The average fluorescence intensity was reported as mean±SEM, however, both average survival time and fluorescence lifetime were reported as mean±SD.

Supplementary figures:

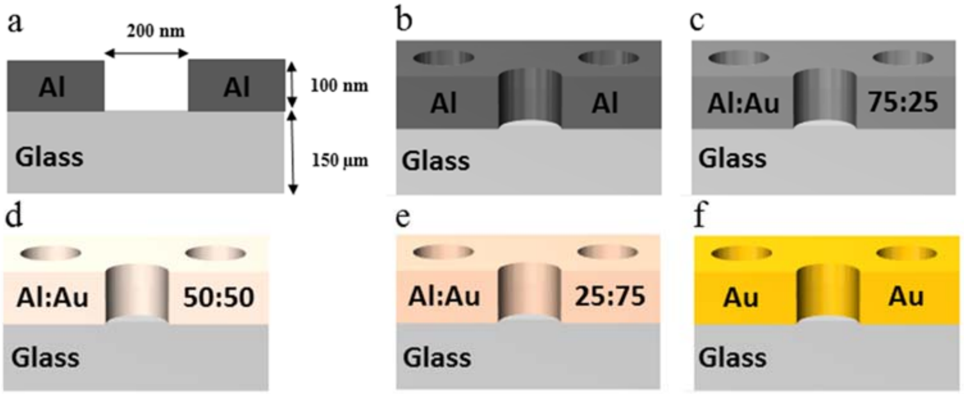

Fig. S1 Schematic of zero mode waveguides with 200 nm diameter holes in a 100 nm thick metal film (a) side view (xz plane) of an aluminum ZMW (b-f) 3-D view of 100Al , 75Al, 50Al, 25Al and 100Au respectively.

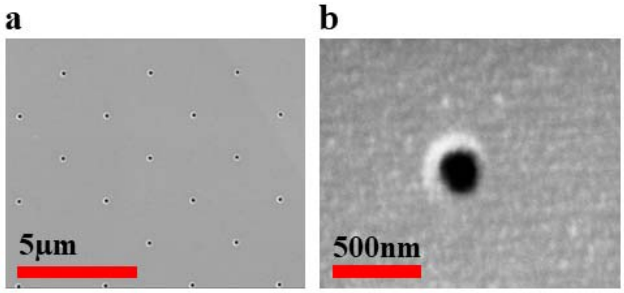

Fig. S2 (a) Scanning electron microscope (SEM) image of an array of ZMW wells showing proper lift off resist polymer and (b) image of a ZMW well at higher magnification.

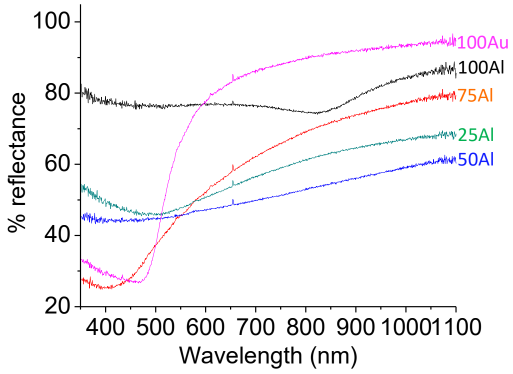

Fig. S3. Diffuse reflectance spectra of 100Al, 75Al, 50Al, 25Al and 100Au. The reflectance spectra of 100 Al and 100 Au matched published results for Al and Au thin films. There is an obvious red shift in the surface plasmon with increased Au content in mixed metal ZMWs. The results presented are semi-quantitative, as the system was not calibrated for absolute determination of % reflectance.

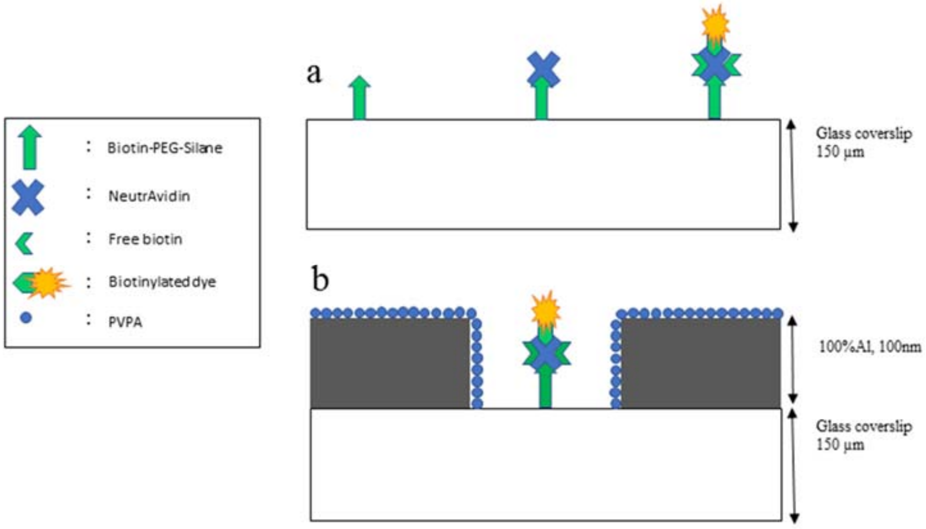

Fig. S4 Immobilization process of ATTO dyes through Biotin-PEG-Silane and neutravidin linker (a) on glass cover slip and (b) on the glass bottom of ZMW well. A layer of Polyvinyl pyrolidone acetate (PVPA) was coated on ZMW walls to prevent non specific binding of ATTO dyes on ZMW walls.

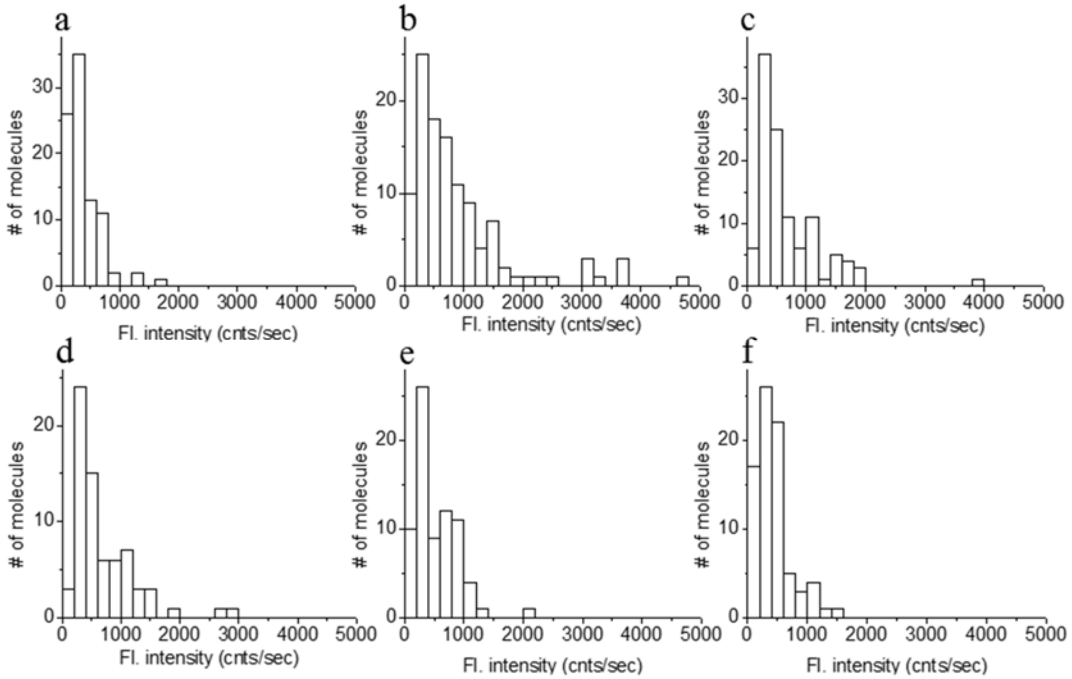

Fig. S5 (a) Fluorescence intensity of single ATTO 550 molecules isolated on glass and (b-f)fluorescence intensity of single ATTO 550 molecules isolated on the glass bottom of 100Al, 75Al, 50Al, 25Al and 100Au respectively.

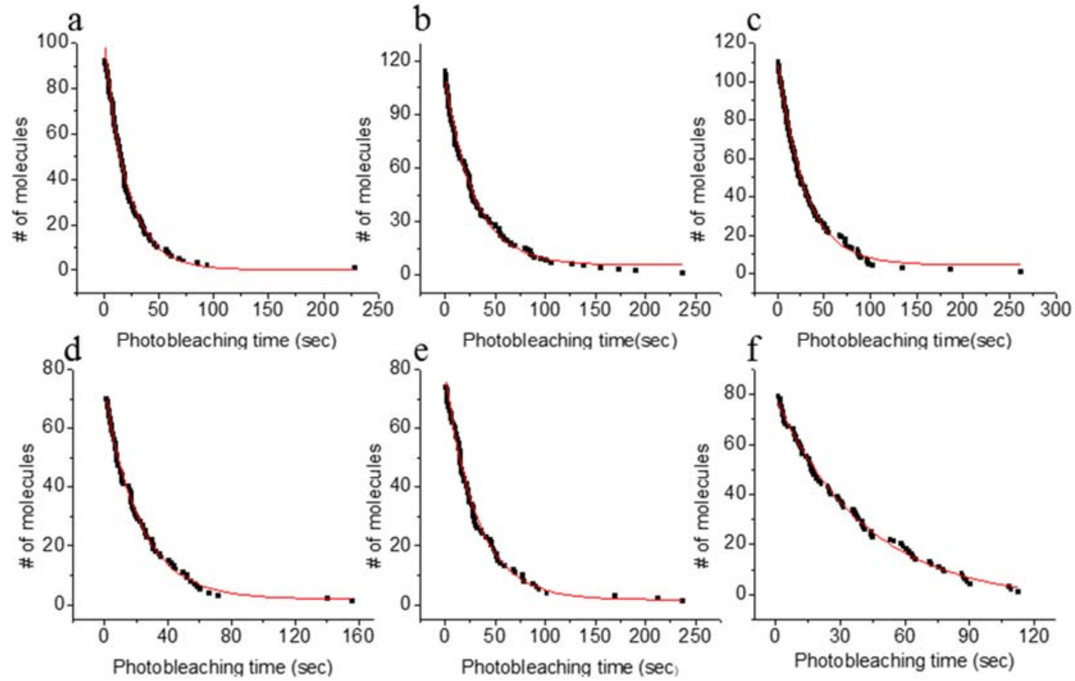

Fig. S6 (a) Survival time of single ATTO 550 molecules isolated on glass and (b-f) survival time of single ATTO 550 molecules isolated on the glass bottom of 100Al, 75Al, 50Al, 25Al and 100Au respectively. Survival times of single ATTO 550 molecules in each substrate were calculated by fitting the photobleaching time of isolated molecules with a single exponential decay.

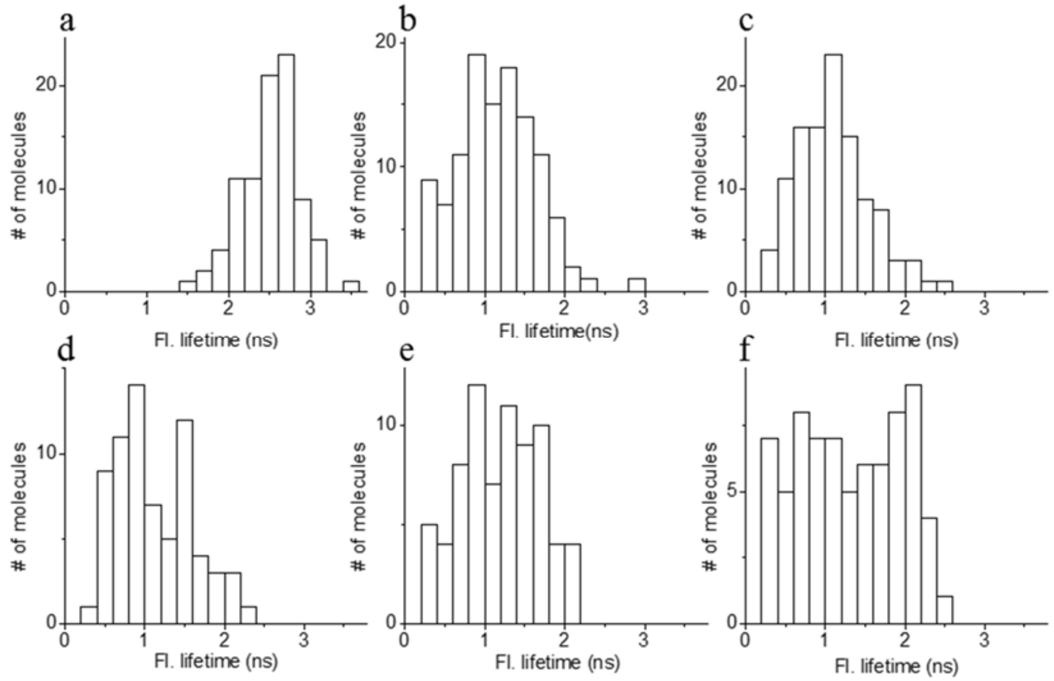

Fig. S7 (a) Fluorescence lifetime of single ATTO 550 molecules isolated on glass and (b-f)fluorescence lifetime of single ATTO 550 molecules isolated on the glass bottom of 100Al, 75Al, 50Al, 25Al and 100Au, respectively.

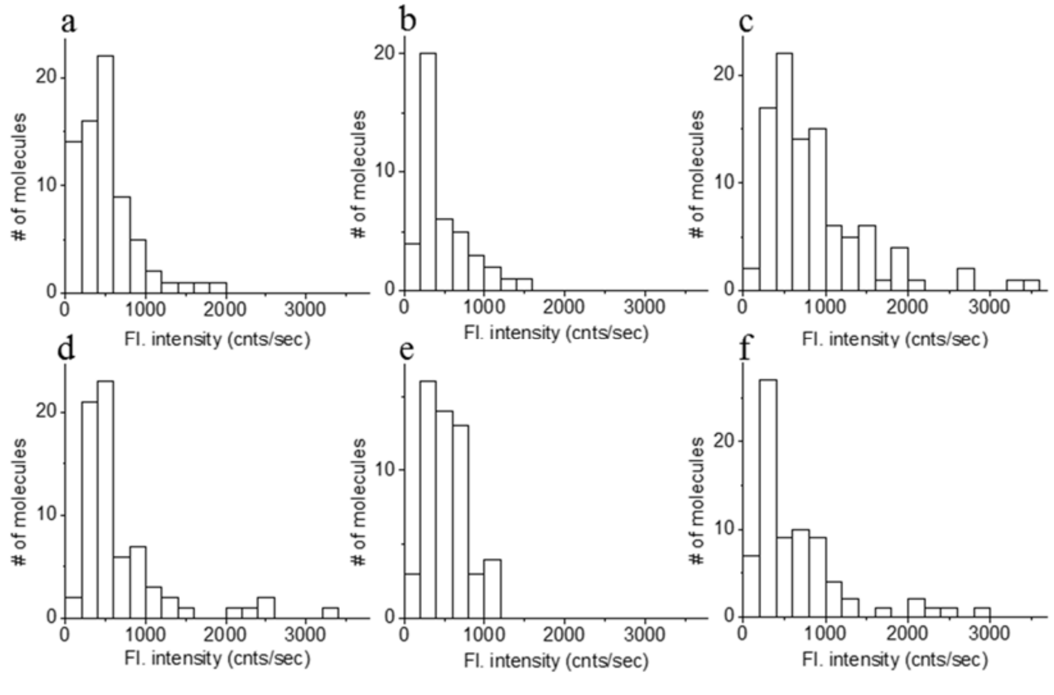

Fig. S8 (a) Fluorescence intensity of single ATTO 590 molecules isolated on glass and (b-f)fluorescence intensity of single ATTO 590 molecules isolated on the glass bottom of 100Al, 75Al, 50Al, 25Al and 100Au, respectively.

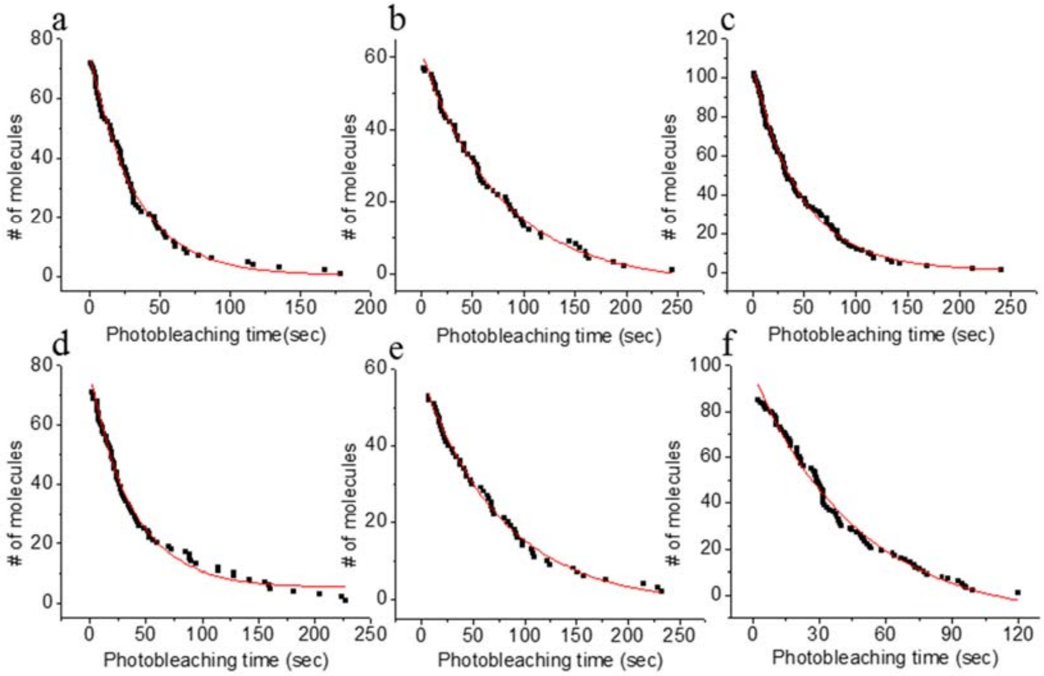

Fig. S9 (a) Survival time of single ATTO 590 molecules isolated on glass and (b-f) survival time of single ATTO 590 molecules isolated on the glass bottom of 100Al, 75Al, 50Al, 25Al and 100Au, respectively. Survival times of single ATTO 590 molecules in each substrate were calculated by fitting the photobleaching time of isolated molecules with a single exponential decay.

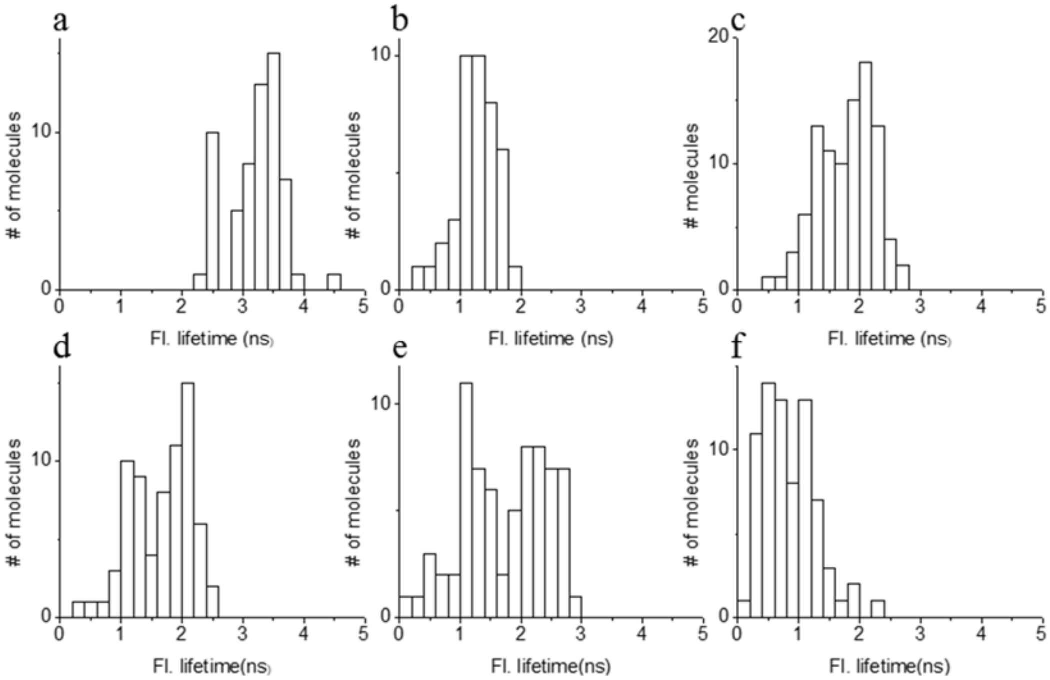

Fig. S10 (a) Fluorescence lifetime of single ATTO 590 molecules isolated on glass and (b-f)fluorescence lifetime of single ATTO 590 molecules isolated on the glass bottom of 100Al, 75Al, 50Al, 25Al and 100Au, respectively.

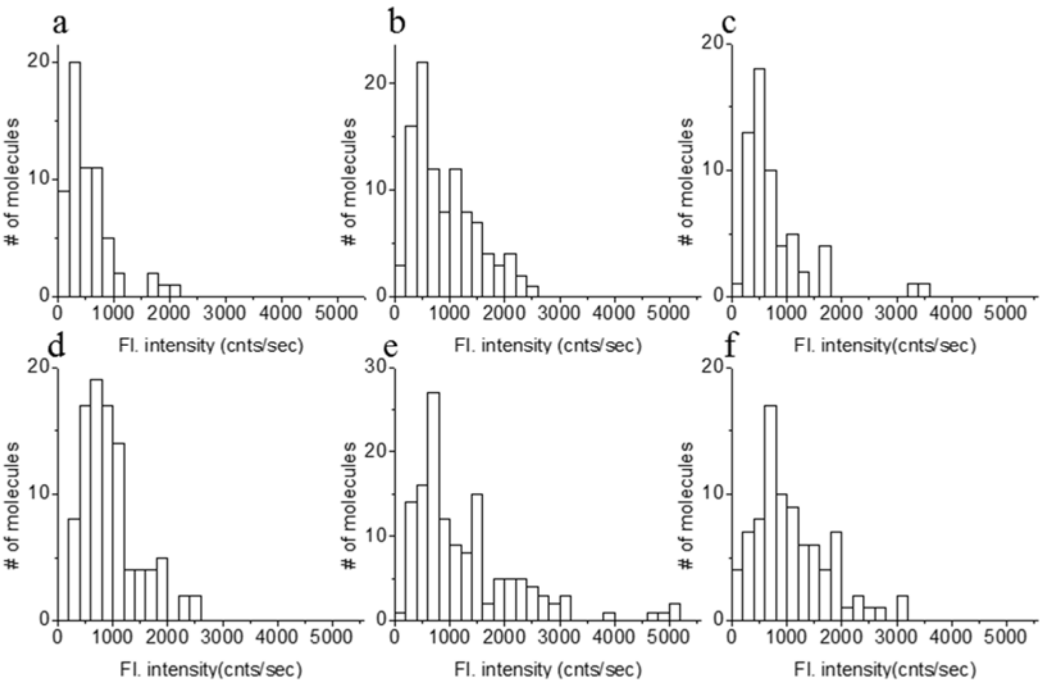

Fig. S11 (a) Fluorescence intensity of single ATTO 610 molecules isolated on glass and (b-f) fluorescence intensity of single ATTO 610 molecules isolated on the glass bottom of 100Al, 75Al, 50Al, 25Al and 100Au, respectively.

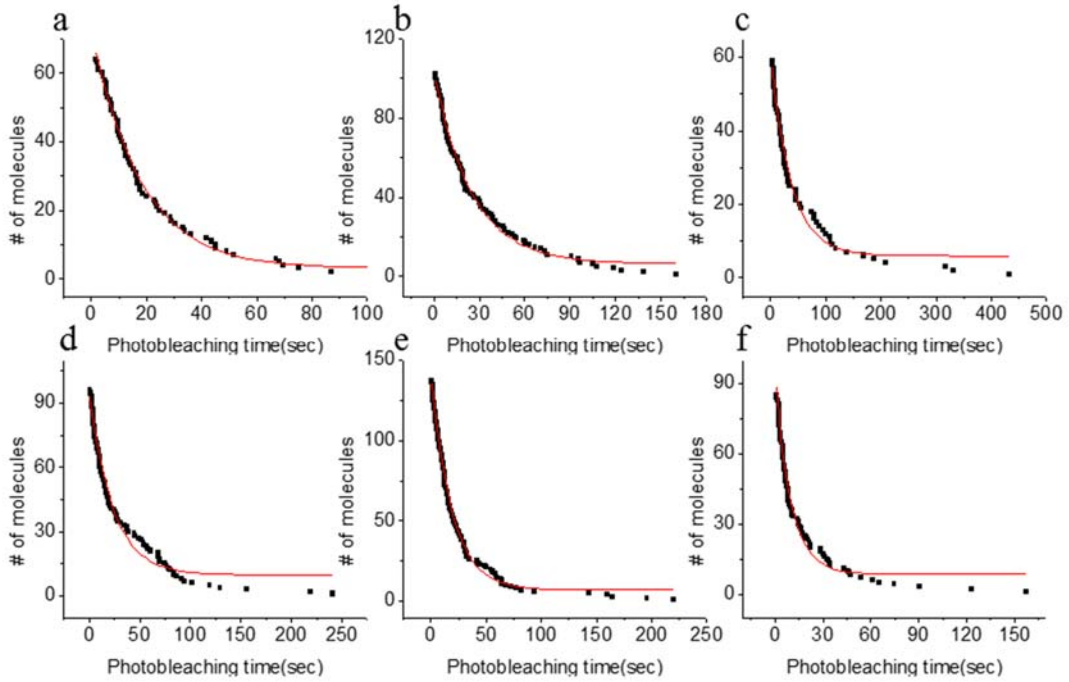

Fig. S12 (a) Survival time of single ATTO 610 molecules isolated on glass and (b-f) survival time of single ATTO 610 molecules isolated on the glass bottom of 100Al, 75Al, 50Al, 25Al and 100Au, respectively. Survival times of single ATTO 610 molecules in each substrate were calculated by fitting the photobleaching time of isolated molecules with a single exponential decay.

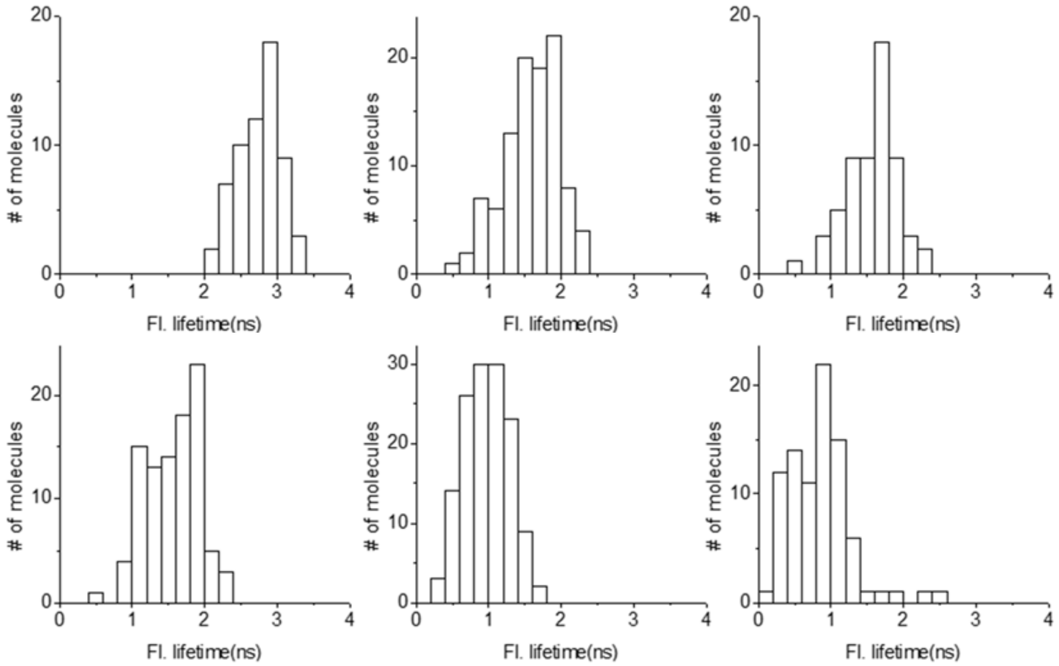

Fig. S13 (a) Fluorescence lifetime of single ATTO 610 molecules isolated on glass and (b-f) fluorescence lifetime of single ATTO 610 molecules isolated on the glass bottom of 100Al, 75Al, 50Al, 25Al and 100Au, respectively.

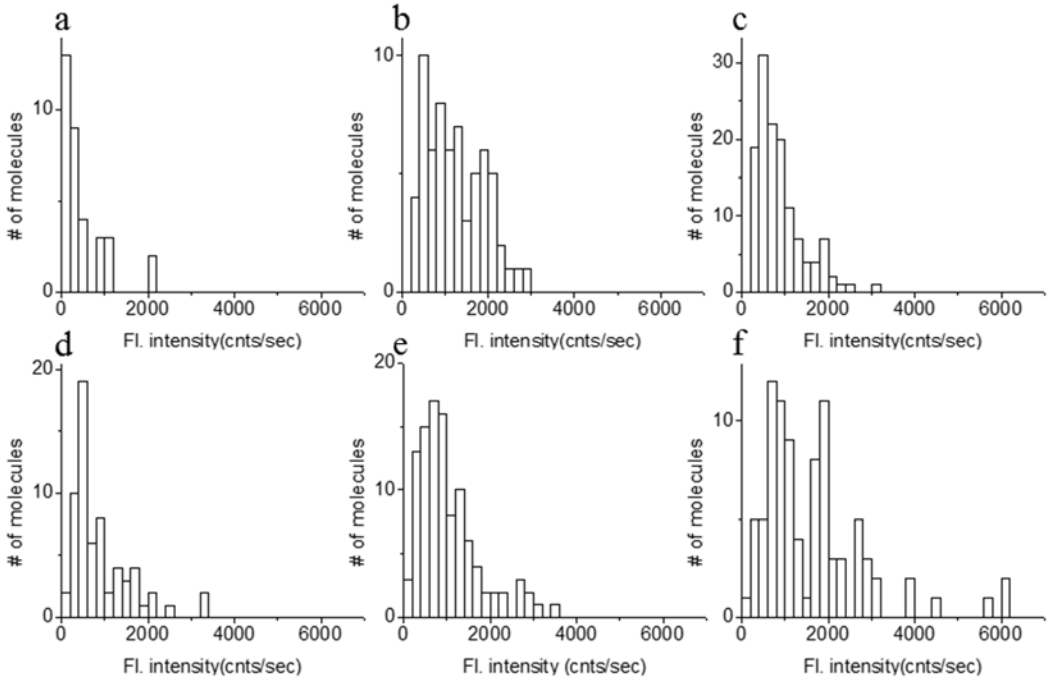

Fig. S14 (a) Fluorescence intensity of single ATTO 647N molecules isolated on glass and (b-f) fluorescence intensity of single ATTO 647N molecules isolated on the glass bottom of 100Al, 75Al, 50Al, 25Al and 100Au, respectively.

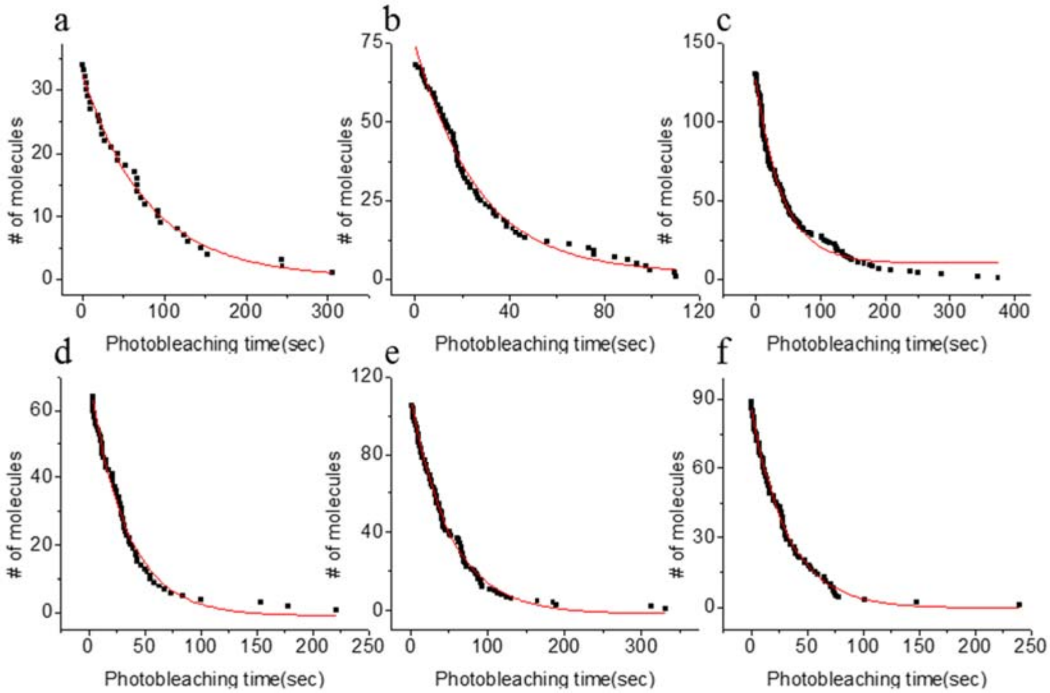

Fig. S15 (a) Survival time of single ATTO 647N molecules isolated on glass and (b-f) survival time of single ATTO 647N molecules isolated on the glass bottom of 100Al, 75Al, 50Al, 25Al and 100Au, respectively. Survival times of single ATTO 647N molecules in each substrate were calculated by fitting the photobleaching time of isolated molecules with a single exponential decay.

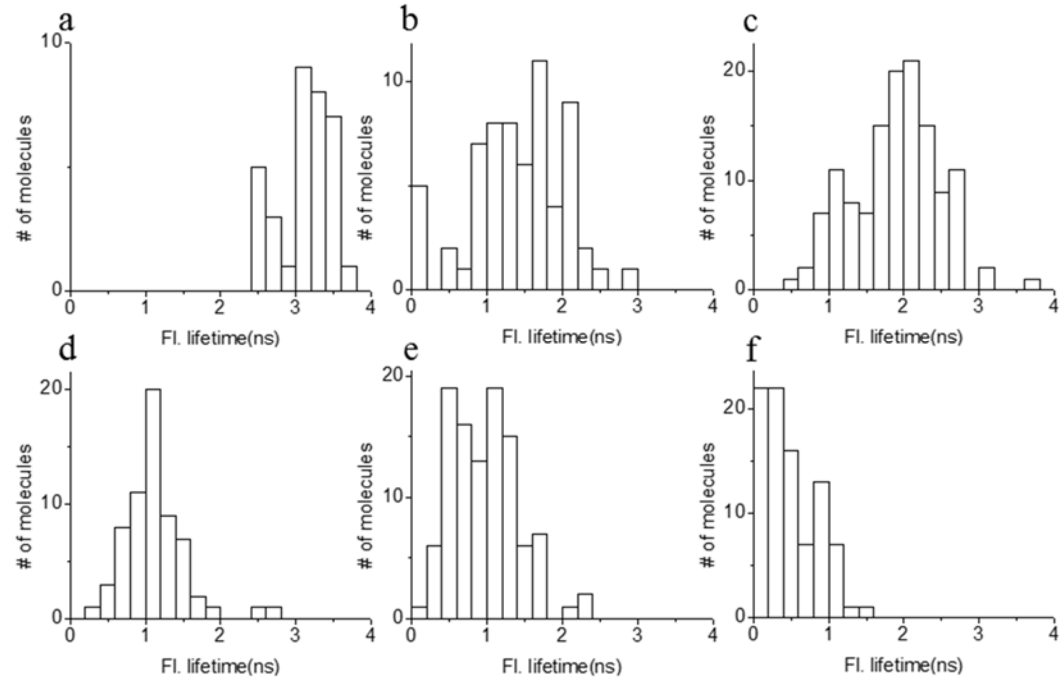

Fig. S16 (a) Fluorescence lifetime of single ATTO 647N molecules isolated on glass and b-f)fluorescence lifetime of single ATTO 647N molecules isolated on the glass bottom of 100Al, 75Al, 50Al, 25Al and 100Au, respectively.

Notes and references

1. W. E. Martin, B. R. Srijanto, C. P. Collier, T. Vosch and C. I. Richards, *The Journal of Physical Chemistry A*, 2016, **120**, 6719-6727.
2. M. Foquet, K. T. Samiee, X. Kong, B. P. Chauduri, P. M. Lundquist, S. W. Turner, J. Freudenthal and D. B. Roitman, *Journal of Applied Physics*, 2008, **103**, 034301.
3. J. Korlach, P. J. Marks, R. L. Cicero, J. J. Gray, D. L. Murphy, D. B. Roitman, T. T. Pham, G. A. Otto, M. Foquet and S. W. Turner, *Proceedings of the National Academy of Sciences*, 2008, **105**, 1176-1181.
